# Association between obesity status and successful aging among older people in China: evidence from CHARLS

**DOI:** 10.1186/s12889-020-08899-9

**Published:** 2020-05-24

**Authors:** Huiqiang Luo, Xiaohui Ren, Jijie Li, Kan Wu, Yixi Wang, Qing Chen, Ningxiu Li

**Affiliations:** 1 West China School of Public Health and West China Fourth Hospital, Sichuan University, Chengdu, No.17 Section 3, Renmin South Road, Chengdu, 610041 Sichuan China; 2 Department of Medical Records, West China Secondary University Hospital, Sichuan University, Chengdu, 610041 Sichuan China; 3 Department of Medical, West China School of Stomatology (West China Hospital of Stomatology), Chengdu, 610041 Sichuan China

**Keywords:** Obesity, Successful aging, Chinese, Older adults, CHARLS

## Abstract

**Background:**

The paper aimed to examine the association between obesity status and successful aging among elderly adults in China and further find gender differences in the effect of components of successful aging on obesity status.

**Methods:**

The data came from the follow-up survey(2015) of China Health and Retirement Longitudinal Study (CHARLS) and 4019 dwellers age 60 and over are included. Obesity status were defined by the body mass index (BMI) according to Chinese criteria. Successful aging was defined following Rowe and Kahn’s multidimensional model. Multivariable logistic regression was used to estimate the relationship between obesity status and successful aging.

**Results:**

The rate of successful aging in men and women was 18.87 and 9.48% respectively. For BMI, the proportion of population with underweight, overweight and obesity in men was 10.29, 23.04 and 29.63% respectively and that in women was 1.40, 11.69 and 9.47%. Men with obesity (OR = 1.587 95% CI 1.087 ~ 2.316) has an positive relationship with successful aging than normal weight men; Women with underweight (OR = 0.197 95% CI 0.058 ~ 0.824) has an negative relationship with successful aging than normal weight women; Meanwhile, no matter men and women, the relationship between obesity status and successful aging were not significant among oldest adults(≥75 years).

**Conclusion:**

Obesity status was significantly association with successful aging in young older adults (60-74 years), and the components of successful aging differently were related with the obesity status of male and female.

## Background

Globally, the number of older persons aged 60 years and over is predicted to increase dramatically from 1.0 billion in 2017 to 3.1 billion by 2100 [1]. Similarly, according to the report of World Health Organization, China, undergoing a faster increase in longevity than expected nowadays, has entered into an aged society and will continue to age rapidly in the future [2].

Naturally, living longer may add the burden to others/family and cause poor quality of life [3]. The evolving of aging’s perspectives have shifted from “how to live longer” to “how to age well” [4]; the definition of successful aging has been perceived as a useful tool for describing the health status of the elderly population since it was put forward [5], Havighurst proposed the early concept of successful aging defined as “getting satisfaction from life” and “adding life to the years” [6]. Rowe and Kahn discussed the operational concept of successful aging that encompasses 3 main components including low risk of disease and disability, maintenance of high physical and cognitive functioning, and active engagement in social and productive activities [7]. Since 1960, there are complementary definitions more or less have been proposed for successful aging, however, there is no unique criterion to define successful aging [8]. Several empirical studies nowadays have recognized the successful aging as a “calculable gold standard of aging” [9]. Though the trend of global aging is increasing dramatically, the rate of successful aging in many counties is still under low level and varied widely. A longitudinal study from Korean found the rate of successful aging in older people(≥65 years) was 10.86% and a national telephone survey on health performed by the French health authorities found 29.9% participants presented good quality of life and qualified as successful aging. A review paper collected 28 researches and found the mean reported rate of successful aging was 35.8% (standard deviation: 19.8; interquartile range: 31%) [4, 8, 10].

Obesity is a global public health challenge [11]. In the US, the prevalence of obesity accounts for one-third of the general population, and another one-third is overweight [12]. Similarly, obesity has become a major public health burden in China [13]. The Chinese older adults also have a higher percentage of body fat than Europeans and U.S. residents with the same BMI [14]. The rates of obesity-related non-communicable diseases (NCDs) in China have also increased extremely and have become the major risk factors for disability and mortality in older adults [15]. Meanwhile, the relationship between obesity and health still exist the gender difference, which may be relevant to the gender difference in obesity. The prevalence of underweight decreased in men but increased in women according to a longitudinal research [16]. And the women are more likely to be obese than men [17].

Moreover, studies reported that older aging is associated with obesity problems, and unhealthy weight change (underweight) [18, 19], which suggests that relevant body weight status could extremely be salient problems among older populations. Obesity status is an essential influence factor for older health [19, 20], as people age, there are some evidences certificate that obesity status is associated with disability [21, 22], cognitive [23], depression [24], chronic disease [25] in older adults. A evidence from European countries found that the epidemic of obesity has a negative influence on life span, and particularly decreases the quality of life during the midlife to older [26]. To date, there are many researches have assessed the association between obesity and older health from a single disease or condition perspectives. Examining a multidimensional construct such as successful aging could provide an improved insight into the association between obesity and overall health.

However, there are little literature to examine the association between obesity status and successful aging, and the results are still controversial. A cross-sectional study found that lower weight was positively associated with successful aging, and obesity negatively influenced aging among 65 to 75 aged French older [8]. A follow-up survey found underweight and obesity were the risk factors for the successful aging among 65 and above older [10]. The evidence from English Longitudinal Study of Ageing indicated that excess BMI was significant associated with shorter healthy and chronic disease-free life expectancy among 50–75 years older. Those researches also have indicated that obesity status may have different relationship with successful aging among different age groups [27]. There are some studies to research the relationship between obesity and successful aging among Chinese, but both of them paid more attention on the demographic status and health behavior, regardless of the health condition such as obesity status [28, 29].

In this paper, the association between obesity status and successful aging was examined among elderly by sex in China using data from CHARLS (China Health and Retirement Longitudinal Study, a longitudinal survey). The present study was prone to assess whether the relationship between obesity status and successful aging was exists, whether the relationship existed the gender difference.

## Methods

### Data

The data in this study were from the follow-up survey of the China Health and Retirement Longitudinal Study (CHARLS). The first national baseline survey and the recent follow-up survey of the CHARLS were fielded in June 2011 and May 2015 respectively. The 2015 survey involved 10,517 respondents aged 60 years old and above. The PPS (probability proportional to scale) and CAPI technology (computer-assisted personal interviewing) were used to randomly choose and face-to-face interview respondents in 28 provinces. The previously research has been published which was used to describe the questionnaire and the quality assurance measures of the survey [30]. The CHARLS study has get the approval for interviewing respondents and collecting data by the Biomedical Ethics Review Committee of Peking University (IRB00001052–11015), and the informed consent was required to sign by the respondents.

**Fig. 1:**
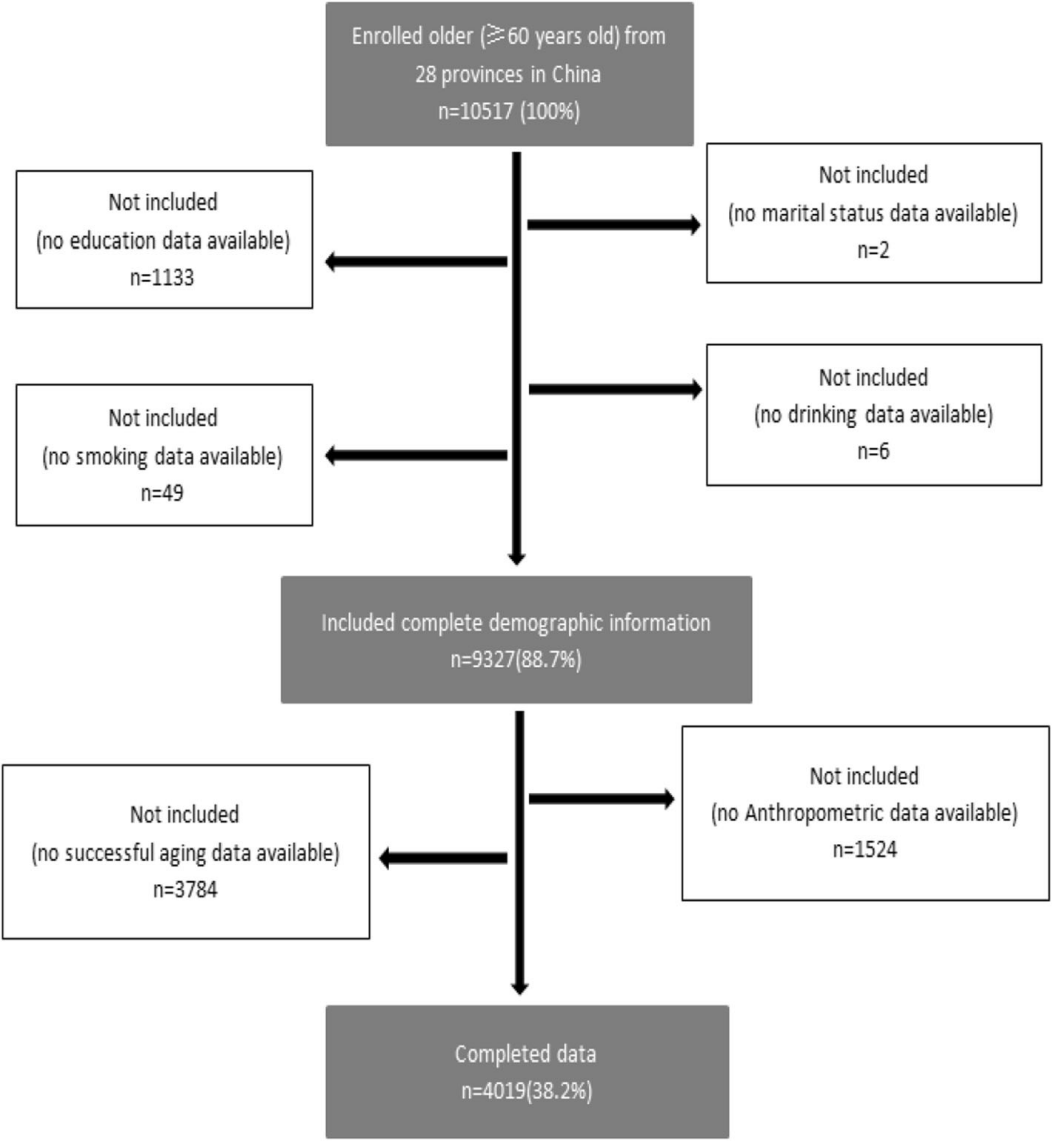
Sample selection Flowchart

This study used the follow-up data from 2015. To research the association between the obesity status and successful aging, we limited the samples to respondents who had received physical examinations in 2015. A total of 4019 subjects were included (Fig. 1).

### Definition of successful aging

As previously mentioned, there is not standard measurement of successful aging exists, and the concept of successful aging differs among cultures. Our criteria for successful aging, according to the definition of Rowe and Kahn [7, 31], include the following five components: 1) absence of major disease, 2) freedom from disability, 3) high cognitive function, 4) no depressive symptoms, 5) active social engagement in life. Therefore, the 5 components mentioned above were included, and participants satisfying all of these conditions were classified into the successful aging group. The single indicator of successful aging is operationalized as follows:

1 Absence of major disease: Conditions of chronic disease were assessed by using the following series of question:“Have you been diagnosed with conditions listed below by a doctor?” The conditions included cancer, chronic lung disease, diabetes, heart disease, and stroke. The research indicated that those disease mentioned above caused the major disease burden for older [32], the Respondents were classified as having no major disease if they reported have no any of the five chronic diseases.

2 Freedom from disability: The ADL scale was used to assess the ADLs [33], according to the following questions: “Because of a physical, mental, emotional or memory problem, do you have any difficulty with one type of everyday activity, excluding any that you expect to last less than three months?” The everyday activities included dressing, bathing or showering, eating, getting into or out of bed, using the toilet, and controlling urination and defecation. Each answer for questions to evaluate impairment in these daily activities was divided into 4 responses as follows: 1) No, I don’t have any difficulty; 2) I have difficulty but can still do it; 3) Yes, I have difficulty and need help; and 4) I cannot do it. In this study, the original answer for each ADLs response was first recorded as 0 if the participant had the ability to complete all of the activities without difficulty, or as 1 if those who had difficulty on any response above. Then a ADL score was derived for each individual to summarize recoded values of all six items. The ADL score was categorized into two groups to reflect degrees of ADL disability: 1) ADL independent (ADL score = 0); 2) Having an ADL disability (ADL score ≥ 1). Respondents were classified as having no disability if they were ADL independent.

3 High cognitive function [2]: Participants were considered to have high cognitive functioning if they achieved a median or higher score using the Telephone Interview for Cognitive Status (TICS). This includes both immediate and delayed recall of ten word son a list, serial subtraction of seven from 100 (up to five times), and naming the day of the week, month, day, year, and season, and drawing the picture.

4 No depressive symptoms: Depressive symptoms were assessed using the CES-D 10(10-item Center for Epidemiological Studies Depression Scale) [34], which has been used for measuring older adults’ depressive symptoms and validated among elderly respondents in China [35], The CES-D 10 scale contains 10 items with response 4 options:1) Rarely or none of the time(< 1 day); 2) Some or a little of the time(1–2 days); 3) Occasionally or a moderate amount of the time(3–4 days); 4) Most or all of the time(5–7 days). The value of 4 options was assigned, ranging from 0 to 3successively. The total of score ranges from 0 to 30, with a lower score indicating a lower level of depressive symptoms. The cut-off score of ≥10 was used to identify the respondents who had depressive symptoms significantly [36].

5 Active social engagement in life: Respondents were defined as being actively social engaged if they participate in any of the following types of social groups: voluntary or charity work, provided help to family, friends, or neighbors, gone to a sport, social, or other kind of club in the month preceding the interview.

### Anthropometric measures

BMI was used to describe general obesity. Respondents were categorized as underweight(<18.5 kg/m2), normal weight (18.5 to 23.9 kg/m2), overweight (24.0 to 27.9 kg/m2), and obese (≥28.0 kg/m2) based on Chinese criteria [13]. These indexes (height and weight) were measured by a stadiometer and scale, respectively (The index of height and weight were collected by the standardized equipment of SecaTM213 Stadiometer and OmronTMHN-286 Scale respectively).

### Socio-demographic and health relevant factors

This study mainly focused on the association between obesity status and successful aging. Previous studies have identified factors that not only had an impact on this relationship but also on obesity status or successful aging alone, such as smoking and drinking, age, sex, education level, marital status, and community type. As a result, these factors were controlled for in this study [3, 4, 37].

### Health-related behavior

The habit of smoking was measured with the question “Have you ever chewed tobacco, smoked a pipe, smoked self-rolled tobacco, or smoked cigarettes/cigars”, and the possible response included the following 3 options: 1) Yes; 2) No; or 3) Quit. The habit of drinking was measured with the question “Did you drink any alcoholic beverages, such as beer, wine, or liquor in the past year, and if so, how often?”, and the possible response included the following: 1) Drink more than once a month; 2) Drink but less than once a month; or 3) Do not drink.

### Socio-demographic characteristics

In this study, the socio-demographic characteristics included age, sex, education level, marital status, and community type. Age was divided into two groups including 60 to 74 (young older) and 75 or older (the oldest). Sex was categorized as male or female. Education level was categorized as illiterate, primary school, or junior high school and above. Marital status was categorized as married, cohabitating and divorced, separated, widowed, or never married. Community type was categorized as urban and rural areas according to the community’s industrial structure; urban areas are generally more developed [36].

### Statistics analysis

Descriptive statistics on individual characteristics were stratified by sex. A chi-squared test was used to compare dichotomous or categorical variables. Multivariable logistic regression analysis was applied to estimate the odds ratio (OR) and 95% confidence interval (CI) for successful aging and its five crucial determining components based on predictor variables. Exhibiting successful aging (1 = successful aging 2 = non successful aging) was a dependent variable. Obesity status (BMI) was independent variables. The regression model included all characteristics as confounding variables including age, sex, education level, marital status, and community type, smoking and drinking. We have examined the interaction between BMI and gender by variance analysis and in order to further determine whether there were associations between successful aging and obesity status between different ages, the interaction between obesity status and age groups, stratified by gender, was tested. The level of statistical significance was set at 95% (*P* < 0.05). Statistical analyses were conducted using SAS 9.3.

## Results

The mean age of the participants was 67.78 (SD = 6.40) years, and 52.48% of the participants were male. The mean age of the enrolled males and females was 67.85 (SD = 6.40) years and 67.69 (SD = 6.41) years respectively. In total, 18.87% (*n* = 398) for males and 9.48% (*n* = 181) for females were defined as values for those who achieved successful aging. Table 1 displays the characteristics of the participants by sex according to the categories of successful aging. Whether males and females, the sample of populations with younger age (60–74; both *P* <0.001), married (males *P* <0.001; females *P* = 0.003), junior high school or above educated (both *P* <0.001), living in urban (both *P* <0.001), keeping low-frequency drinking habits (drink but less than once a month; males *P* = 0.008; females *P* = 0.001) in this study has more higher proportion of successful aging.

**Table 1:** Sample Socio-demographic Characteristics according to successful aging by sex

	Male (N(%))			Female(N(%))		
	Successful Aging	No Successful Aging	*P* Value	Successful Aging	No Successful Aging	*P* Value
**Total**	398(18.87)	1711(81.13)	NA	181(9.48)	1729(90.52)	NA
**Demographic**						
**Age (year)**						
60 ~ 64	363(20.46)	1411(79.54)	< 0.001	167(10.38)	1442(89.62)	< 0.001
≥ 75	35(10.45)	300(89.55)		14(4.65)	287(95.35)	
**Marital status**						
Married/cohabitating	369(20.02)	1474(79.98)	< 0.001	159(10.51)	1354(89.49)	0.003
Divorced/separated/widowed/never married	29(10.90)	237(89.10)		22(5.54)	375(94.46)	
**Socio-economic status**						
**Education level**						
Illiterate	15(4.95)	288(95.05)	< 0.001	25(2.68)	908(97.32)	< 0.001
Primary school and below	164(14.88)	938(85.12)		87(11.85)	647(88.15)	
Junior high school or above	219(31.11)	485(68.89)		69(28.40)	174(71.60)	
**Community type**						
Rural	235(14.07)	1435(85.93)	< 0.001	118(7.25)	1510(92.75)	< 0.001
Urban	163(37.13)	276(62.87)		63(22.34)	219(77.66)	
**Health behavior**						
**Smoking**						
Yes	206(18.23)	924(81.77)	0.553	8(6.78)	110(93.22)	0.077
No	84(18.63)	367(81.37)		172(9.90)	1565(90.10)	
Quit	108(20.45)	420(79.55)		1(1.82)	54(98.18)	
**Drinking**						
Drink more than once a month	201(20.20)	794(79.80)	0.008	11(6.36)	162(93.64)	0.001
Drink but less than once a month	50(24.39)	155(75.61)		21(19.44)	87(80.56)	
Do not drink	147(16.17)	762(83.83)		149(9.15)	1480(90.85)	

As Table 2 shows, the association between obesity status and successful aging and its specific components by sex was analyzed. The proportion of males who were classified as “successful aging “ according to the obesity status categories were 10.29, 15.88, 23.04, and 29.63% for people who were underweight, normal, overweight, obese, respectively. The proportion of females was 1.40, 9.13, 11.69, and 9.47%, respectively. Overall, the association between the distribution of specific components comprising successful aging and obesity status was significant regardless of gender, excluding the “active social engagement in life” in female.

**Table 2:** Association between obesity status and successful aging and its specific components by sex

Successful aging	Male (N,(%))					Female (N,(%))				
	Underweight	Normal	Overweight	Obese	*P* Value	Underweight	Normal	Overweight	Obese	*P* Value
**Successful aging**										
Yes	398(10.29)	184(15.88)	144(23.04)	56(29.63)	< 0.001	2(1.40)	76(9.13)	76(11.69)	27(9.47)	0.002
No	1711(89.71)	975(84.12)	481(76.96)	133(70.37)		141(98.60)	756(90.87)	574(88.31)	258(90.53)	
**Absence of major disease**										
Yes	122(89.71)	1083(93.44)	584(93.44)	166(87.83)	0.020	132(92.31)	772(92.79)	587(90.31)	249(87.37)	0.035
No	14(10.29)	76(6.56)	41(6.56)	23(12.17)		11(7.69)	60(7.21)	63(9.69)	36(12.63)	
**Freedom from disability**										
Yes	92(67.65)	862(74.37)	524(83.84)	147(77.78)	< 0.001	55(38.46)	482(57.93)	403(62.00)	157(55.09)	< 0.001
No	44(32.35)	297(25.63)	101(16.16)	42(22.22)		88(61.54)	350(42.07)	247(38.00)	128(44.91)	
**High cognitive function**										
Yes	56(41.18)	577(49.78)	383(61.28)	120(63.49)	< 0.001	23(16.08)	245(29.45)	235(36.15)	101(35.44)	< 0.001
No	80(58.82)	582(50.22)	242(38.72)	69(36.51)		120(83.92)	587(70.55)	415(63.85)	184(64.56)	
**No depressive symptoms**										
Yes	86(63.24)	836(72.13)	486(77.76)	162(85.71)	< 0.001	66(46.15)	485(58.29)	407(62.62)	187(65.61)	< 0.001
No	50(36.76)	323(27.87)	139(22.24)	27(14.29)		77(53.85)	347(41.71)	243(37.38)	98(34.39)	
**Active social engagement in life**										
Yes	62(45.59)	544(46.94)	334(53.44)	102(53.97)	0.026	64(44.76)	408(49.04)	313(48.15)	153(53.68)	0.294
No	74(54.41)	615(53.06)	291(46.56)	87(46.03)		79(55.24)	424(50.96)	337(51.85)	132(46.32)	

Tables 3 and 4 display the OR values of obesity status and successful aging and its components by gender. For the male, significant association between obesity status and successful aging and its components was observed. Compared with normal weight population, people with overweight has an positive relationship with successful aging (OR = 1.586, 95% CI = 1.243 ~ 2.025), and more prone to be high cognitive function (OR = 1.596, 95% CI = 1.310 ~ 1.946), no depressive symptoms (OR = 1.351, 95% CI = 1.075 ~ 1.697), active social engagement (OR = 1.298, 95% CI = 1.068 ~ 1.577), however, after multivariate adjustment the differences were small and failed to reach statistical significance. After adjusting for confounding factors, compared with normal weight population, the differences between the obese people and major disease, cognitive function failed to reach statistical significance, but significant differences in successful aging and depression, the obese population were significant associated with successful aging (OR = 1.587, 95% CI = 1.087 ~ 2.316) and depression (OR = 1.979, 95% CI = 1.278 ~ 3.063).

**Table 3:** OR(95% CI) of obesity status of successful aging and its components in the Male elderly population

Successful aging	Obesity status (OR (95% CI))			
	Normal	Underweight	Overweight	Obese
**Successful aging**				
Unadjusted Model	1.000	0.608(0.342–1.081)	1.586(1.243–2.025)^***^	2.231(1.573–3.165)^***^
Adjusted Model^a^	1.000	0.701(0.387–1.269)	1.172(0.902–1.522)	1.587(1.087–2.316)^*^
**Absence of major disease**				
Unadjusted Model	1.000	0.612(0.336–1.114)	1.000(0.675–1.481)	0.506(0.309–0.830)^**^
Adjusted Model	1.000	0.616(0.335–1.134)	1.184(0.790–1.776)	0.653(0.391–1.092)
**Freedom from disability**				
Unadjusted Model	1.000	0.939(0.584–1.510)	1.053(0.806–1.377)	0.938(0.622–1.415)
Adjusted Model	1.000	1.135(0.699–1.844)	0.952(0.720–1.260)	0.826(0.538–1.268)
**High cognitive function**				
Unadjusted Model	1.000	0.706(0.492–1.012)	1.596(1.310–1.946)^***^	1.754(1.277–2.410)^**^
Adjusted Model	1.000	0.786(0.528–1.170)	1.171(0.938–1.461)	1.201(0.841–1.715)
**No depressive symptoms**				
Unadjusted Model	1.000	0.665(0.458–0.963)^*^	1.351(1.075–1.697)^*^	2.318(1.512–3.554)^***^
Adjusted Model	1.000	0.727(0.499–1.061)	1.171(0.925–1.484)	1.979(1.278–3.063)^**^
**Active social engagement in life**				
Unadjusted Model	1.000	0.947(0.663–1.353)	1.298(1.068–1.577)^**^	1.325(0.974–1.804)
Adjusted Model	1.000	1.024(0.711–1.474)	1.150(0.937–1.410)	1.160(0.840–1.602)

^a^Adjusted for age, marital status, education level, community type, smoking, drinking. *p < 0.05;**p < 0.01;***p < 0.001. *OR* Odds ratio, *CI* confidence interval

**Table 4:** OR(95% CI) of obesity status of successful aging and its components in the Female elderly population

Successful aging	Obesity status (OR (95% CI))			
	Normal	Underweight	Overweight	Obese
**Successful aging**				
Unadjusted Model	1.000	0.141(0.034–0.581)^**^	1.317(0.941–1.843)	1.041(0.656–1.651)
Adjusted Model^a^	1.000	0.197(0.047–0.824)^*^	1.181(0.828–1.686)	0.942(0.578–1.534)
**Absence of major disease**				
Unadjusted Model	1.000	0.933(0.478–1.820)	0.724(0.500–1.048)	0.538(0.347–0.832)^**^
Adjusted Model	1.000	0.951(0.485–1.865)	0.716(0.493–1.038)	0.520(0.334–0.811)^**^
**Freedom from disability**				
Unadjusted Model	1.000	0.522(0.356–0.766)^**^	0.974(0.759–1.249)	0.640(0.473–0.867)^**^
Adjusted Model	1.000	0.568(0.384–0.839)^**^	0.902(0.700–1.162)	0.574(0.420–0.784)^***^
**High cognitive function**				
Unadjusted Model	1.000	0.459(0.287–0.735)^**^	1.357(1.090–1.688)^**^	1.315(0.989–1.749)
Adjusted Model	1.000	0.624(0.371–1.048)	1.240(0.956–1.607)	1.249(0.889–1.754)
**No depressive symptoms**				
Unadjusted Model	1.000	0.613(0.429–0.876)^**^	1.198(0.971–1.479)	1.365(1.031–1.807)^*^
Adjusted Model	1.000	0.645(0.450–0.925)^*^	1.153(0.932–1.427)	1.309(0.984–1.741)
**Active social engagement in life**				
Unadjusted Model	1.000	0.842(0.589–1.203)	0.965(0.786–1.185)	1.205(0.920–1.577)
Adjusted Model	1.000	0.877(0.611–1.258)	0.946(0.768–1.164)	1.231(0.936–1.620)

^a^Adjusted for age, marital status, education level, community type, smoking, drinking. *p < 0.05;**p < 0.01;***p < 0.001. *OR* Odds ratio, *CI* confidence interval

For the female, after adjusting for confounders, those, compared with normal weight people, who were underweight population had odds ratios of 0.141 (95% CI = 0.034 ~ 0.581) for successful aging, 0.522(95% CI = 0.356 ~ 0.766) for “freedom from disability”, 0.645(95% CI = 0.450 ~ 0.925) for “no depressive symptoms”, but multivariate adjusted analyses showed that underweight people had no significant difference in “high cognitive function” (OR = 0.624, 95% CI = 0.371 ~ 1.048) compared with normal weight people. Meanwhile, the multivariate adjusted analyses also displayed that obese people had odds ratios of 0.520 (95% CI = 0.334 ~ 0.811) for “absence of major disease”, 0.574 (95% CI = 0.420 ~ 0.784) for “freedom from disability” compared with normal weight population.

Meanwhile, we found the interaction between obesity status and gender has significant effect on successful aging (F = 4.879 *P* < 0.001). To further determine whether obesity status has different association with successful aging between different age groups, we also tested the association between obesity status and successful aging among different age groups stratified by sex (Table 5). Compared to young old men (60–74 years) with normal weight, the young old men (60–74 years) with obese weight had odds ratios of 0.607 (*p* < 0.05), whereas, there were no significant differences in successful aging among the oldest men (75 and above years) regardless of obesity status. As for women, there existed significant differences in successful aging among the young older (60–74 years) with odds ratios of 3.998 (*p* < 0.05) for underweight, compared with normal weight.

**Table 5:** Interaction of obesity status and age on successful aging stratified by gender

Obesity status	Male (Odds ratios)^**b**^		Female (Odds ratios)	
	60 ~ 74	≥75	60 ~ 75	≥75^a^
Normal	1.000	1.000	1.000	NA
Underweight	0.670	0.535	0.180^*^	NA
Overweight	1.527	1.751	1.34	NA
Obese	2.197^***^	1.284	1.105	NA

Note. ^a^Both of the population with ≥75 years in female cannot reach the criteria of the successful aging, as a result, the analysis isn’t include the oldest(≥75 years) in female. ^b^Odds ratios were estimated by multivariate logistic regression model adjusted for marital status, education level, community type, smoking and drinking. *p < 0.05, ***p < 0.001

## Discussion

Our study found a significant association between obesity status and successful aging among different sex by using the analysis of national representative sample for older adults in China. The result showed that the rate of successful aging in male was 18.87%, with 10.29, 15.88, 23.04 and 29.63% reporting underweight, normal weight, overweight and obese respectively and those for female was 9.48%, with 1.40, 9.13, 11.69 and 9.47% reporting underweight, normal weight, overweight and obese respectively. Meanwhile, gender differences in obesity status were found across components of successful aging (major disease, disability, cognitive function, depression and social engagement).

In terms of males, compared with the older adults who were normal weight, the obese population was significantly associated with successful aging in male (OR = 1.587 95% CI = 1.087 ~ 2.316), and the association between obese and other 4 components except depression (OR = 1.979 95% CI = 1.278 ~ 3.063) was not significant. Our findings about the relationship between obesity status and depression in male was consistent with previous epidemic studies focusing on elderly people [13, 36, 38].

Our prior study found that Obesity is more likely to relate with onset of depression in men, and the men with obesity were less likely to have depressive symptoms than normal weight men according to the follow up data [13]. The inverse association between obesity status and depression can be explained by the applying of “Jolly fat” hypothesis [39], the men are prone to judge a smaller body frame to be less preferable than a larger, more muscular one [40]; Chinese people are more likely to have positive perceptions of obesity because it is considered as acquiring good fortune to become fat during middle age in traditional Chinese culture [41]. We also could conclude that the inverse association between obesity status and successful aging in male may result from the association between obesity status and depression.

In terms of females, the underweight population was less likely to achieve successful aging (OR = 0.197 95% CI = 0.047 ~ 0.824) compared with normal weight. Specifically speaking, women with underweight was more likely to suffer from depression (OR = 0.645 95% CI = 0.450 ~ 0.925) than normal weight women, which was consistent with previous studies. Lee found that underweight was associated with the increased risk for depression, this pattern of relationship was more prominent in female and young older adults than male and elderly adults [42], they found the unfavorable socioeconomic or medical conditions inducing underweight can be a major cause of depression, eating or behavior disorders following depression can lead underweight in turn. Moreover, our findings that female with low BMI (underweight) was at a high ADL risk of OR = 0.568 (95% CI = 0.384 ~ 0.839), was consistent with published literatures performed on older adults [43–46].

Obese women were more likely to suffer from major disease (OR = 0.520 95% CI = 0.334 ~ 0.811) than normal weight women, our findings were consistent with most existing studies that obesity made contributes to a higher of many chronic diseases, such as CVD, musculoskeletal disease, and diabetes [47]. Similarly, the high BMI (obese) also was negatively associated with ADLs (OR = 0.574 (95% CI = 0.420 ~ 0.784)), Obesity was verified with increased risk of disabilities [48–50], moreover, some studies also indicated that obesity has become a serious problem in people with disabilities those were less likely to take exercise [51], in other words, there was an adverse circle between disability and obesity. Yang and colleagues found that the effect of obesity on ADL disability might be independent from the obesity-related chronic disease [46].

To further determine whether obesity status has different associations with successful aging between different age groups, this paper also tested for interaction between obesity status and age groups stratified by gender (Table 5). In the group of younger older people (60 ~ 74), as for male, the young obese older adults (60 ~ 74) had odds ratios of 2.197 for to be non-successful aging compared with normal weight population; Compared to normal weight young older adults (60 ~ 74) in female, the underweight population had odds ratios of 0.180 for to be non-successful aging. In the group of oldest adult (≥75), there was no significant association between obesity status and successful aging among male, and we cannot find similar trend result from the lack of successful female older. The reasons may explain this phenomenon as follow: First, the BMI is associated with the risk for all-cause mortality among younger older people (65 ~ 74), however, no enough evidence can support similar correlation among people aged 75 and above years old [52]. Second, the criteria of successful aging are relative strict and there is a little number of participants who are one of oldest adult (≥75) and can achieve successful aging relatively, compared with younger older people (60 ~ 74) (the rate of successful aging is 15.7% in younger older people and that is 7.7% in oldest adult). Third, the proportion of people who are overweight, underweight or obese is relatively lower. As a result, the sample size may cause the non-significant result and bias, it is also one of the limitations, the follow-up research may be helpful.

Our study has some limitations.

First, the CESD-10 is still a commonly tool to evaluate clinically significant depressive symptoms, which is a reasonable and well-established method in epidemiological study, but there is still a concern that CESD-10 may overestimate or underestimate depression.

Second, gender disparities would be occur leading to misclassification in reporting symptoms, because there were studies proved that female is more likely to report depression and disability than male [53, 54].

Third, the cross-sectional study cannot conclude causal inference between BMI and successful aging, the follow-up in the future can support persuaded evidence.

Furthermore, the result cannot be generalized to other ethnic groups, because the study adopted the Chinese criteria of obesity status.

## Conclusion

In conclusion, this cross-sectional study found that obesity status was significantly association with successful aging in young older adults (60-74 years), and the components of successful aging differently were related with the obesity status of male and female.

In terms of male, the obese population was significant associated with successful aging compared with normal weight population after adjusting relevant confounders, which may be relation with the association between obesity status and depression (obese population was less likely to suffer from the onset of depression). In terms of female, compared to normal weight population, underweight people was less likely to achieve successful aging, speaking specifically, the risk of ADL disability and depression was higher for female with underweight.

## Data Availability

The dataset collected and analyzed in the current study are available from the corresponding author on reasonable request.

## Abbreviations

CHARLS: China Health and Retirement Longitudinal Study
BMI: Body mass index
ADL Scale: Activity of Daily Living Scale
CES-D 10: 10-item Center for Epidemiological Studies Depression Scale
